# Impact of Preoperative Type 2 Diabetes Mellitus on the Outcomes of Gastric Cancer Patients Following Gastrectomy: A Propensity Score Matching Analysis

**DOI:** 10.3389/fsurg.2022.850265

**Published:** 2022-03-08

**Authors:** Yu-Xi Cheng, Wei Tao, Bing Kang, Xiao-Yu Liu, Chao Yuan, Bin Zhang, Dong Peng

**Affiliations:** ^1^Department of Gastrointestinal Surgery, The First Affiliated Hospital of Chongqing Medical University, Chongqing, China; ^2^Department of Clinical Nutrition, The First Affiliated Hospital of Chongqing Medical University, Chongqing, China

**Keywords:** gastric cancer, type 2 diabetes mellitus, propensity score matching, outcome, prognosis

## Abstract

**Purpose:**

The current study aims to explore the outcomes of type 2 diabetes mellitus (T2DM) on gastric cancer patients following gastrectomy through propensity score matching (PSM) analysis.

**Methods:**

A retrospective study of gastric cancer patients following gastrectomy was conducted in a single clinical center from January 2014 to December 2019. The short-term outcomes, overall survival (OS) and disease-free survival (DFS) were analyzed between T2DM group and Non-T2DM group.

**Results:**

A total of 703 patients were enrolled in this study. After 1:1 PSM, 84 patients in T2DM group and 84 patients in Non-T2DM were matched for final analysis. No significant difference was found in terms of operation time, intra-operative blood loss, retrieved lymph nodes, postoperative stay, blood transfusion and complications between T2DM group and Non-T2DM group (*p* > 0.05). The Kaplan-Meier curve implied that T2DM had no impact on OS or DFS. Cox regression was conducted to identify predictive factors for prognosis. Body mass index (BMI) (*p* = 0.039 < 0.05, HR = 0.725, 95% CI = 0.534–0.983), pre-operative lymphocyte (*p* = 0.017 < 0.05, HR = 0.678, 95% CI = 0.493–0.932), pathological tumor node metastasis (pTNM) stage (*p* = 0.000 < 0.05, HR = 2.619, 95% CI = 2.048–3.349) and complications (*p* = 0.006 < 0.05, HR = 1.528, 95% CI = 1.132–2.061) were predictive factors for OS, and BMI (*p* = 0.013 < 0.05, HR = 0.524, 95% CI = 0.315–0.872), pTNM stage (*p* = 0.000 < 0.05, HR = 2.619, 95% CI = 2.048–3.349) and complications (*p* = 0.008 < 0.05, HR = 1.892, 95% CI = 1.179–3.036) were independent predictive factors for DFS.

**Conclusion:**

T2DM did not have an impact on gastric cancer patients following gastrectomy in terms of short-term outcomes and prognosis.

## Introduction

Gastric cancer is the fifth most common cancer worldwide and the third leading cause of cancer-related deaths (1, 2). More than one million gastric cancer cases were newly diagnosed worldwide each year, of which 44% were diagnosed in China (1, 3). Although the expanding application of multidisciplinary team boomed in gastric cancer, surgery is the only curing method for resectable cases (4, 5).

Type 2 diabetes mellitus (T2DM) is a metabolic disease with a high incidence worldwide, and it is also one of the leading causes of death in the world (6). As was reported, 493 million people were affected by T2DM in 2019, and 700 million people would develop T2DM in 2045 (7, 8).

It has been reported that T2DM could increase the incidence of gastric cancer (9, 10), however, the impact of T2DM on prognosis after gastrectomy remained controversial (11, 12). The propensity score matching (PSM) method could reduce the interference caused by the mismatch of baseline information (13). However, there were few studies using PSM analysis to explore the effect of T2DM on gastrectomy (12, 14). Therefore, the current study aims to explore the outcomes of T2DM on gastric cancer patients following gastrectomy through PSM.

## Methods

### Patients

A retrospective study of gastric cancer patients following gastrectomy was conducted in a single clinical center from January 2014 to December 2019, which was carried out in accordance with the World Medical Association Declaration of Helsinki. This study was reviewed and approved by the Institutional Review Board of local hospital (2021-336) and all the patients signed informed consent.

### Inclusion and Exclusion Criteria

**Figure 1:**
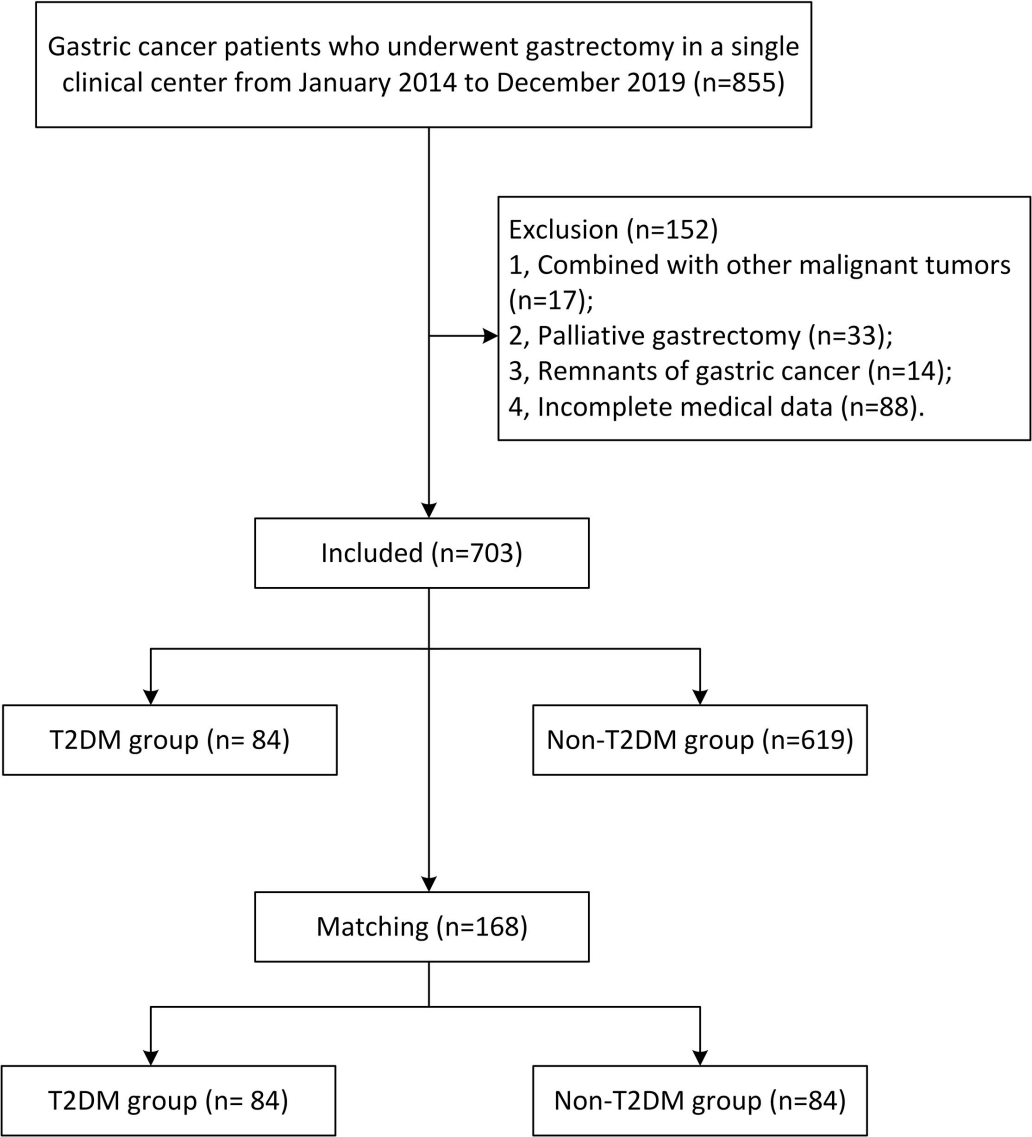
Flow-chart of patient selection.

Gastric cancer patients who underwent gastrectomy were included in this study (*n* = 855), and the patients were excluded by the following criteria: 1, combined with other malignant tumors (*n* = 17); 2, palliative gastrectomy (*n* = 33); 3, remnants of gastric cancer (14); and 4, incomplete medical data (*n* = 88). Finally, a total of 703 patients were included in this study, and the flow of selection was shown in Figure 1.

### Surgery Management

Patients were confirmed gastric cancer pathologically before surgery, and underwent gastrectomy plus D2 lymph node dissection according to the Japanese gastric cancer treatment guidelines (15). All the surgeries were conducted by two surgeons who had more than 10 years' experience in a team. Patients were regularly followed up every 3 months in the first 2 years and every 6 months in the following 3 years.

### Definitions

We classified the postoperative complications in accordance with the Clavien-Dindo classification (16). Overall survival (OS) was defined as the time from gastrectomy to death or last follow-up, and disease-free survival (DFS) was defined as the time from gastrectomy to recurrence, death or last follow-up.

The remission of T2DM was divided into three situations: complete remission, partial remission and no remission (17, 18). Complete remission was defined as follows: fasting blood glucose (FBG) returned to a normal range with no requirement for medication. Partial remission was defined as follows: FBG returned to an improved level with reduced medication. No remission was defined as follows: no changes in medication, more medication requirements or aggravation in FBG levels after surgery.

### Data Collection

The perioperative information was retrospectively collected through outpatient and inpatient system including baseline information, surgical information and postoperative information. The baseline information included age, sex, body mass index (BMI), T2DM duration, anti-diabetes medication, T2DM status after gastrectomy, neo-adjuvant chemotherapy, pre-operative hemoglobin, pre-operative albumin, pre-operative lymphocyte; The surgical information included type of resection, surgery methods, reconstruction methods, operation time, intra-operative blood loss and retrieved lymph nodes; The postoperative information included pathological stage, complications, blood transfusion and postoperative hospital stay; The follow-up information included OS and DFS which was conducted by inpatient system and telephone interviews.

### PSM

To minimize the selection bias of patients, PSM was conducted between T2DM group and Non-T2DM group. Nearest neighbor matching was performed without replacement at a 1:1 ratio and a caliper width with a 0.01 standard deviation was specified. The baseline information was matched including age, sex, BMI, neo-adjuvant chemotherapy, pre-operative hemoglobin, pre-operative albumin, type of resection, surgery methods, reconstruction methods and pathological stage.

### Statistical Analysis

Continuous variables are expressed as the mean ± SD, and independent-sample *t*-test was used to compare the difference between T2DM group and Non-T2DM group. Frequency variables are expressed as n (%), and Chi-square test or Fisher's exact test was used. The Kaplan-Meier curve was conducted to compare T2DM on different pathological stage, and cox regression analyses were performed to identify predictive factors for OS and DFS. Data were analyzed using SPSS (version 25.0) statistical software. A bilateral *p*-value of < 0.05 was considered statistically significant.

## Results

### Patient Information Before PSM

A total of 703 patients were enrolled in the current study according to the inclusion and exclusion criteria. There were 84 T2DM patients and 619 Non-T2DM patients. The clinical characteristics of the 84 T2DM patients including age, BMI, sex, combined hypertension, T2DM duration, anti-diabetes medication and T2DM status after gastrectomy were concluded in Table 1. In this study, 47.6% of patients had T2DM remission after gastrectomy.

**Table 1:** Clinical characteristics of patients with concurrent gastric cancer and T2DM.

**Characteristics**	**No. 84**
Age (years)	64.1 ± 9.7
BMI (kg/m^2^)	23.5 ± 3.3
**Sex**	
Male	60 (71.4%)
Female	24 (28.6%)
Combined hypertension	36 (42.9%)
**T2DM duration**	
<5 years	
≥ 5 years	37 (44.0%) 47 (56.0%)
**Anti-diabetes**	
Oral hypoglycemic agent only	68 (81.0%)
Insulin (with or without oral hypoglycemic agent)	16 (19.0%)
**T2DM status after gastrectomy**	
No remission	44 (52.4%)
Partial remission	24 (28.6%)
Complete remission	16 (19.0%)

*Variables are expressed as the mean ± SD, n (%)*.

*T2DM, type 2 diabetes mellitus; BMI, body mass index*.

Besides, the patient information including age, sex, BMI, neo-adjuvant chemotherapy, pre-operative hemoglobin, pre-operative albumin, type of resection, surgery methods, reconstruction methods and pathological stage were compared between the two groups. T2DM group had higher age (*p* = 0.004 < 0.05), BMI (*p* = 0.000 < 0.05), open surgery (*p* = 0.014 < 0.05), pre-operative hemoglobin (*p* = 0.008 < 0.05), and pre-operative albumin (*p* = 0.043 < 0.05). No significant difference was found in type of resection, reconstruction methods and pathological stage (*p* > 0.05) (Table 2).

**Table 2:** Baseline characteristics before propensity score matching.

**Characteristics**	**T2DM (84)**	**Non-T2DM (619)**	**t/χ^2^ value**	***P*-value**
Age (years)	64.1 ± 9.7	60.6 ± 10.6	−2.856	0.004*
Sex			0.009	0.923
Male	60 (71.4%)	439 (70.9%)		
Female	24 (28.6%)	180 (29.1%)		
BMI (kg/m^2^)	23.5 ± 3.3	22.1 ± 3.1	−3.881	0.000*
Neo-adjuvant chemotherapy	5 (6.0%)	35 (5.7%)	0.012	0.805
Pre-operative hemoglobin (g/L)	112.5 ± 30.9	121.1 ± 27.5	2.643	0.008*
Pre-operative albumin (g/L)	38.3 ± 6.4	39.8 ± 6.1	2.032	0.043*
Type of resection			0.457	0.499
Subtotal gastrectomy	56 (66.7%)	435 (70.3%)		
Total gastrectomy	28 (33.3%)	184 (29.7%)		
Open surgery	6 (7.1%)	12 (1.9%)	8.029	0.014*
Reconstruction methods			1.913	0.384
B-I	33 (39.3%)	290 (46.8%)		
B-II	17 (20.2%)	120 (19.4%)		
R-Y	34 (40.5%)	209 (33.8%)		
pTNM stage			5.620	0.060
I	21 (25.0%)	193 (31.2%)		
II	14 (16.7%)	149 (24.1%)		
III	49 (58.3%)	277 (44.7%)		

Variables are expressed as the mean ± SD, n (%), *P < 0.05.

*T2DM, type 2 diabetes mellitus; BMI, body mass index; B-I, Billroth I reconstruction; B-II, Billroth II reconstruction; R-Y, Roux-en-Y reconstruction; pTNM, pathological tumor node metastasis*.

### PSM Analysis

There were differences between T2DM group and Non-T2DM group, therefore, PSM was conducted to minimize the difference. After 1:1 PSM, 84 patients in T2DM group and 84 patients in Non-T2DM were matched for final analysis. There was no difference in terms of age, sex, BMI, neo-adjuvant chemotherapy, pre-operative hemoglobin, pre-operative albumin, type of resection, surgery methods, reconstruction methods and pathological stage (*p* > 0.05) (Table 3).

**Table 3:** Baseline characteristics after propensity score matching.

**Characteristics**	**T2DM (84)**	**Non-T2DM (84)**	**t/χ^2^ value**	***P*-value**
Age (year)	64.1 ± 9.7	62.9 ± 9.5	−0.863	0.389
Sex			0.029	0.865
Male	60 (71.4%)	59 (70.2%)		
Female	24 (28.6%)	25 (29.8%)		
BMI (kg/m^2^)	23.5 ± 3.3	23.5 ± 3.2	−0.002	0.998
Neo-adjuvant chemotherapy	5 (6.0%)	2 (2.4%)	1.342	0.443
Pre-operative hemoglobin (g/L)	112.5 ± 30.9	111.1 ± 30.3	−0.290	0.772
Pre-operative albumin (g/L)	38.3 ± 6.4	38.6 ± 5.8	0.339	0.735
Type of resection			1.244	0.265
Subtotal gastrectomy	56 (66.7%)	49 (58.3%)		
Total gastrectomy	28 (33.3%)	35 (41.7%)		
Open surgery	6 (7.1%)	4 (4.8%)	0.425	0.514
Reconstruction methods			0.267	0.875
B-I	33 (39.3%)	32 (38.1%)		
B-II	17 (20.2%)	15 (17.9%)		
R-Y	34 (40.5%)	37 (44.0%)		
pTNM stage			4.673	0.097
I	21 (25.0%)	14 (16.7%)		
II	14 (16.7%)	25 (29.8%)		
III	49 (58.3%)	45 (53.5%)		

*Variables are expressed as the mean ± SD, n (%)*.

*T2DM, type 2 diabetes mellitus; BMI, body mass index; B-I, Billroth I reconstruction; B-II, Billroth II reconstruction; R-Y, Roux-en-Y reconstruction; pTNM, pathological tumor node metastasis*.

### Short-Term Outcomes

We compared the operation time, intra-operative blood loss, retrieved lymph nodes, postoperative stay, blood transfusion and complications between T2DM group and Non-T2DM group after PSM. However, no significant difference was found (*p* > 0.05) (Table 4).

**Table 4:** Short-term outcomes after propensity score matching.

**Characteristics**	**T2DM (84)**	**Non-T2DM (84)**	**t/χ^2^ value**	***P*-value**
Operation time (minutes)	223.0 ± 65.1	247.9 ± 106.4	1.828	0.069
Intra-operative blood loss (ml)	137.5 ± 158.5	190.8 ± 360.0	−0.851	0.396
Retrieved lymph nodes	19.5 ± 9.9	21.7 ± 9.2	1.489	0.138
Postoperative hospital stay (days)	13.4 ± 7.8	14.9 ± 18.2	0.732	0.465
Blood transfusion	14 (16.7%)	7 (8.3%)	2.667	0.102
Overall complications	24 (28.6%)	27 (32.1%)	0.253	0.615

*Variables are expressed as the mean ± SD, n (%)*.

*T2DM, type 2 diabetes mellitus*.

### Prognosis

**Figure 2:**
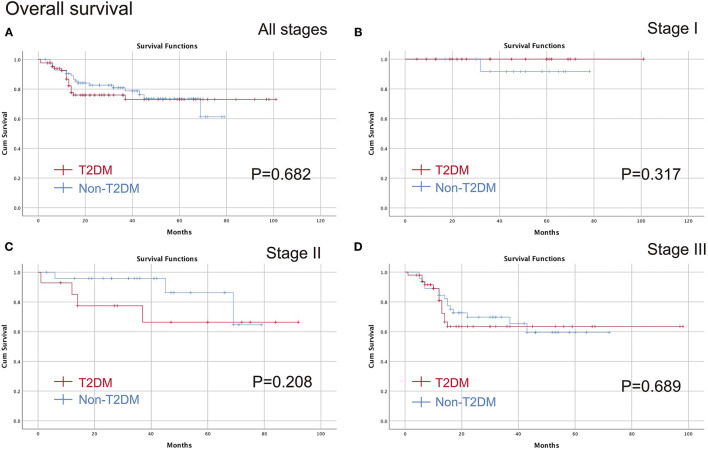
Comparison of the OS between T2DM group and Non-T2DM group. **(A)** all stages; **(B)** stage I; **(C)** stage II; **(D)** stage III. OS, overall survival; T2DM, type 2 diabetes mellitus.

**Figure 3:**
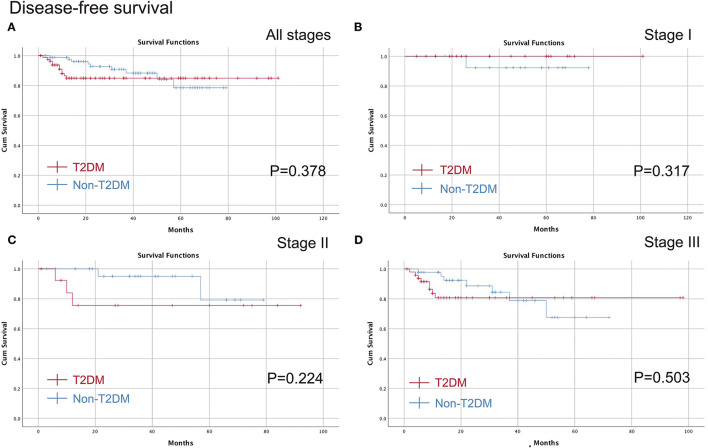
Comparison of the DFS between T2DM group and Non-T2DM group. **(A)** all stages; **(B)** stage I; **(C)** stage II; **(D)** stage III. DFS, disease free survival; T2DM, type 2 diabetes mellitus.

The medium follow-up time was 29 (1–101) months. The Kaplan-Meier curve was conducted to compare T2DM on different pathological stages. No significant difference was found in all stages (*p* = 0.682), stage I (*p* = 0.317), stage II (*p* = 0.208) or stage III (*p* = 0.689) in terms of OS, and furthermore, there were no differences in all stages (*p* = 0.378), stage I (*p* = 0.317), stage II (*p* = 0.224) or stage III (*p* = 0.503) in terms of DFS (Figures 2, 3).

### Cox Regression

Cox regression was conducted to identify predictive factors for prognosis. In terms of OS, BMI (*p* = 0.039 < 0.05, HR = 0.725, 95% CI = 0.534–0.983), pre-operative lymphocyte (*p* = 0.017 < 0.05, HR = 0.678, 95% CI = 0.493–0.932), pathological tumor node metastasis (pTNM) stage (*p* = 0.000 < 0.05, HR = 2.619, 95% CI = 2.048–3.349) and complications (*p* = 0.006 < 0.05, HR = 1.528, 95% CI = 1.132–2.061) were predictive factors. In terms of DFS, BMI (*p* = 0.013 < 0.05, HR = 0.524, 95% CI = 0.315–0.872), pTNM stage (*p* = 0.000 < 0.05, HR = 2.619, 95% CI = 2.048-3.349) and complications (*p* = 0.008 < 0.05, HR = 1.892, 95% CI = 1.179–3.036) were independent prognostic factors. In addition, T2DM was not the predictive factor for OS (*p* = 0.595 > 0.05, HR = 0.876, 95% CI = 0.538–1.426) or DFS (*p* = 0.211 > 0.05, HR = 1.509, 95% CI = 0.792–2.874) (Tables 5, 6).

**Table 5:** Univariate and multivariate analysis of overall survival.

**Risk factors**	**Univariate analysis**		**Multivariate analysis**	
	**HR (95% CI)**	***P*-value**	**HR (95% CI)**	***P*-value**
Age (years) (>/ ≤ 62.0)	1.326 (0.993–1.771)	0.056		
Sex (female/male)	1.028 (0.747–1.414)	0.867		
BMI (kg/m^2^) (>/ ≤ 22.2)	0.629 (0.468–0.846)	0.002*	0.725 (0.534–0.983)	0.039*
T2DM (yes/no)	0.876 (0.538–1.426)	0.595		
Pre-operative hemoglobin (g/L) (>/ ≤ 125.0)	0.583 (0.433–0.786)	0.000*	0.890 (0.637–1.243)	0.495
Pre-operative albumin (g/L) (>/ ≤ 41.0)	0.580 (0.423–0.795)	0.001*	0.808 (0.573–1.139)	0.223
Pre-operative lymphocyte (10^9^/L) (>/ ≤ 1.4)	0.534 (0.394–0.722)	0.000*	0.678 (0.493–0.932)	0.017*
Type of resection (Subtotal gastrectomy/Total gastrectomy)	2.432 (1.818–3.252)	0.000*	1.403 (0.790–2.490)	0.248
Open surgery (yes/no)	1.399 (0.657–2.979)	0.384		
Reconstruction methods (R-Y/B-II/B-I)	1.597 (1.356–1.881)	0.000*	1.071 (0.777–1.477)	0.674
Complications (yes/no)	1.831 (1.367–2.450)	0.000*	1.528 (1.132–2.061)	0.006*
Blood transfusion (yes/no)	1.896 (1.260–2.853)	0.002*	1.220 (0.794–1.876)	0.365
pTNM stage (III/II/I)	3.055 (2.407–3.877)	0.000*	2.619 (2.048–3.349)	0.000*

*P < 0.05.

*HR, Hazard ratio; CI, confidence interval; BMI, body mass index; T2DM, type 2 diabetes mellitus; B-I, Billroth I reconstruction; B-II, Billroth II reconstruction; R-Y, Roux-en-Y reconstruction; pTNM, pathological tumor node metastasis*.

**Table 6:** Univariate and multivariate analysis of disease-free survival.

**Risk factors**	**Univariate analysis**		**Multivariate analysis**	
	**HR (95% CI)**	***P*-value**	**HR (95% CI)**	***P*-value**
Age (years) (>/ ≤ 62.0)	1.094 (0.686–1.745)	0.706		
Sex (female/male)	0.857 (0.502–1.463)	0.571		
BMI (kg/m^2^) (>/ ≤ 22.2)	0.491 (0.301–0.804)	0.005*	0.524 (0.315–0.872)	0.013*
T2DM (yes/no)	1.509 (0.792–2.874)	0.211		
Pre-operative hemoglobin (g/L) (>/ ≤ 125.0)	0.626 (0.389–1.008)	0.054		
Pre-operative albumin (g/L) (>/ ≤ 41.0)	0.562 (0.337–0.935)	0.027*	0.694 (0.415–1.161)	0.164
Pre-operative lymphocyte (10^9^/L) (>/ ≤ 1.4)	0.622 (0.386–1.001)	0.050		
Type of resection (Subtotal gastrectomy/Total gastrectomy)	2.429 (1.520–3.881)	0.000*	1.044 (0.426–2.557)	0.924
Open surgery (yes/no)	2.271 (0.828–6.229)	0.111		
Reconstruction methods (R-Y/B-II/B-I)	1.653 (1.268–2.155)	0.000*	1.288 (0.780–2.127)	0.323
Complications (yes/no)	2.244 (1.408–3.576)	0.001*	1.892 (1.179–3.036)	0.008*
Blood transfusion (yes/no)	1.853 (0.949–3.618)	0.071		
pTNM stage (III/II/I)	3.760 (2.453–5.762)	0.000*	2.619 (2.048–3.349)	0.000*

*P < 0.05.

*HR, Hazard ratio; CI, confidence interval; BMI, body mass index; T2DM, type 2 diabetes mellitus; B-I, Billroth I reconstruction; B-II, Billroth II reconstruction; R-Y, Roux-en-Y reconstruction; pTNM, pathological tumor node metastasis*.

## Discussion

A total of 703 patients were included in this study. After 1:1 PSM, 84 patients in T2DM group and 84 patients in Non-T2DM group were matched for final analysis. No significant difference was found in terms of operation time, intra-operative blood loss, retrieved lymph nodes, postoperative stay, blood transfusion, complications, OS or DFS between T2DM group and Non-T2DM group. Furthermore, BMI, pre-operative lymphocyte, pTNM stage and complications were predictive factors for OS, and BMI, pTNM stage and complications were predictive factors for DFS.

T2DM is a metabolic disease, which is related to the onset of digestive tumors including gastric cancer, colorectal cancer, and esophageal cancer (9, 19, 20). In addition to the influence on tumor onset, T2DM might also affect the prognosis of patients, including OS, DFS, and cancer-specific survival (12, 14). A study reported that the OS of gastric cancer patients could be improved by metformin (21). Among patients with gastric cancer, there was also another study reporting that the remission of T2DM would affect the prognosis as well (22). There were few studies concerning about the postoperative outcomes and prognosis through PSM, therefore, the current study aims to explore the outcomes of T2DM on gastric cancer patients following gastrectomy through PSM.

A recent study reported that T2DM was associated with increased infection and readmission rate (23). Lee et al. (24) reported T2DM could increase the postoperative complications, postoperative stay, combined with other diseases and the status of hyperglycemia might contribute to the complications. However, in our study, it was found no statistical difference in terms of postoperative stay or complications, which was consistent with previous studies (25).

The mechanism between T2DM and gastric cancer remained unclear, some potential mechanism might contribute to the tumor genesis: 1. The status of hyperglycemia which promoted the tumor cells proliferation (26); 2. In addition, T2DM together with obesity is a chronic low-grade inflammatory disease which could result in malignant tumors (27); and 3. The mitosis and metastasis of tumor cells were enhanced in hyperinsulinemia (28).

Chen et al. (12) reported T2DM could decrease the progress-free survival. In another study, Sheng et al. (14) reported T2DM could apparently worsen the OS of gastric cancer patients undergoing gastrectomy. However, in the PSM study, it was not statistically significant in terms of OS or DFS. Therefore, large-scale prospective studies should be conducted to determine the exact results of T2DM on prognosis.

In this study, BMI, pre-operative lymphocyte, tumor stage and complications were predictive factors of OS. Besides, BMI, pTNM stage and complications were predictive factors for DFS. The results were consisting with previous studies (29, 30). Pre-operative lymphocyte was closely associated with human's immune function, and lower level of lymphocyte indicated worse OS in this study (31, 32). Therefore, perioperative management should be careful to conduct to improve the outcomes of gastric patients.

Few studies reported the prognosis of T2DM on gastric cancer patients following gastrectomy (12, 14). To our knowledge, this is the first study to analyze the short-term outcomes of T2DM on gastric cancer patients using PSM. Inconsistent with the previous studies, T2DM had no effect on short-term outcomes or prognosis in this study.

Furthermore, there was an interesting phenomenon that FBG level improved after gastrectomy in some patients concurrent with gastric cancer and T2DM, and the conclusion was proved previously (11, 33, 34). The remission rate of T2DM in our single center was 47.6%, which was approximately consistent with the previous studies (17, 18, 35–37). Gastrectomy, regarded as the onco-metabolic surgery, could control metabolic diseases including hypertension and T2DM (38, 39). This phenomenon was found in CRC patients as well (19, 40). However, the influence on remission of T2DM after gastrectomy needed more prospective studies to prove in the future.

There were several limitations in this present study as well. First, it could bring some selection bias due to the retrospective single-center study with a small amount of cases; Second, the medium follow-up time was relatively short; Third, we were lacking the data related diabetes severity, such as glycated hemoglobin, C-peptide and degree of diabetic complication; Last, some nutritional or immunological factors including neutrophil-lymphocyte ratio, platelet-lymphocyte ratio, CD4^+^/ CD8^+^, IL-18 and IL-1β (41–44) were lacking for further analysis. Therefore, some large scale and multi-center prospective randomized controlled trials with more comprehensive data should be conducted in the following experiments.

In conclusion, T2DM did not have an impact on gastric cancer patients following gastrectomy in terms of short-term outcomes and prognosis.

## Data Availability Statement

The raw data supporting the conclusions of this article will be made available by the authors, without undue reservation.

## Ethics Statement

The studies involving human participants were reviewed and approved by the Ethics Committee of The First Affiliated Hospital of Chongqing Medical University, 2021-336. The patients/participants provided their written informed consent to participate in this study.

## Author Contributions

WT, BK, X-YL, CY, and BZ: data extraction. DP: data analysis. DP, Y-XC, and WT: quality assessments. Y-XC: writing—original draft. DP, Y-XC, WT, BK, X-YL, CY, and BZ: writing—review and editing. All authors have read and approved the final manuscript.

## Conflict of Interest

The authors declare that the research was conducted in the absence of any commercial or financial relationships that could be construed as a potential conflict of interest.

## Publisher's Note

All claims expressed in this article are solely those of the authors and do not necessarily represent those of their affiliated organizations, or those of the publisher, the editors and the reviewers. Any product that may be evaluated in this article, or claim that may be made by its manufacturer, is not guaranteed or endorsed by the publisher.
